# Social Ecology and Diabetes Self-Management among Pacific Islanders in Arkansas

**DOI:** 10.23937/2469-5793/1510026

**Published:** 2016-03-14

**Authors:** Pearl Anna McElfish, Ramey Moore, David Woodring, Rachel S. Purvis, Gregory G. Maskarinec, Williamina Ioanna Bing, Jonell Hudson, Peter O. Kohler, Peter A. Goulden

**Affiliations:** 1 Office of Community Health and Research, University of Arkansas for Medical Sciences Northwest, USA; 2 Department of Anthropology, University of Arkansas, USA; 3 Family Medicine and Community Health, University of Hawai’i at Manoa, USA; 4 Pharmacy Practice, University of Arkansas for Medical Sciences Northwest, USA; 5 University of Arkansas for Medical Sciences Northwest, USA; 6 College of Medicine, University of Arkansas for Medical Sciences, USA

**Keywords:** Health disparities, Community-based participatory research, Pacific Islanders, Minority health, Diabetes type 2

## Abstract

Chronic diseases disproportionately affect ethnic and racial minorities. Pacific Islanders, including the Marshallese, experience some of the highest documented rates of type 2 diabetes. Northwest Arkansas is home to the largest population of Marshallese outside of the Republic of the Marshall Islands, and many migrants are employed by the local poultry industry. This migrant population continues to increase because of climate change, limited health care and educational infrastructure in the Marshall Islands, and the ongoing health effects of US nuclear testing. The US nuclear weapons testing program had extensive social, economic, and ecological consequences for the Marshallese and many of the health disparities they face are related to the nuclear fallout. Beginning in 2013, researchers using a community-based participatory (CBPR) approach began working with the local Marshallese community to address diabetes through the development and implementation of culturally appropriate diabetes self-management education in a family setting. Preliminary research captured numerous and significant environmental barriers that constrain self-management behaviors. At the request of our CBPR stakeholders, researchers have documented the ecological barriers faced by the Marshallese living in Arkansas through a series of qualitative research projects. Using the Social Ecological Model as a framework, this research provides an analysis of Marshallese health that expands the traditional diabetes self-management perspective. Participants identified barriers at the organizational, community, and policy levels that constrain their efforts to achieve diabetes self-management. We offer practice and policy recommendations to address barriers at the community, organizational, and policy level.

## Introduction

Chronic diseases, such as heart disease, overweight/obesity, and type 2 diabetes, disproportionately affect ethnic and racial minorities [1–3]. Pacific Islanders, including Marshallese from the Republic of the Marshall Islands, experience some of the highest documented rates of type 2 diabetes–ranging from 25% to 50% for Marshallese adults–compared to the total US population (8.6%) [4–6].

Springdale, Arkansas is home to the largest population of Marshallese migrants in the continental United States, and migration from the Republic of the Marshall Islands to the United States continues to grow due to climate change, limited health care and education infrastructure, and ongoing health effects from US nuclear testing in the Republic of the Marshall Islands [7,8]. The poultry industry in northwest Arkansas is the primary employer of Marshallese migrants, and access to poultry industry jobs encourages Marshallese migration to the area [9]. In order to explain the context of the diabetes epidemic in the Marshallese community, it is important to understand the historical relationship between the Republic of the Marshall Islands and the United States.

Between 1946 and 1958, the US military detonated 67 fission and thermonuclear devices on Marshall Island atolls, which were equivalent to 7,200 Hiroshima-sized bombs. The largest of these detonations–a single 15 megaton hydrogen bomb over 1,000 times the power of the Hiroshima bomb–occurred on Bikini Atoll, exposing Marshallese to widespread radioactive fallout [10,11]. The nuclear weapons testing program had extensive social, economic, and ecological consequences for the Marshallese. There are many health disparities related to the nuclear fallout, including disruptions in the native food supply and lifestyle [10,12]. After the nuclear testing, the United States sent high-calorie, low-nutrient, processed foods to the Republic of the Marshall Islands as food aid [13]. US food aid transformed the Marshallese diet and many Marshallese still rely on and prefer these unhealthy foods. In addition to nuclear testing, a growing body of literature suggests the prolonged and rising rates of obesity and diabetes among Pacific Islander populations are exacerbated by the adoption of Western lifestyles, and a lack of access to health care and to health education [14,15].

The United States had trusteeship of the Marshall Islands beginning in 1947, and in 1986, the Republic of the Marshall Islands became an independent nation through the signing of a Compact of Free Association (COFA). Under the COFA agreement, Marshallese from the Republic of the Marshall Islands are allowed to freely enter and work in the United States; however, their COFA migrant status limits their access to federal assistance programs, such as Medicaid.

The United States continues to derive numerous benefits from the COFA, including the use of 11 of the 97 islands of Kwajalein Atoll for military activity, which allows the US military to maintain control of approximately two million square miles of the Pacific. While the US military continues to benefit from the COFA, Marshallese migrants struggle to access basic health care services both in the United States and in the Republic of the Marshall Islands.

In 2013, researchers from the University of Arkansas for Medical Sciences Northwest campus began working with the Marshallese community using a community-based participatory research (CBPR) approach to address the health disparities affecting the Marshallese community. Marshallese stakeholders identified diabetes as a primary concern, and the lead researcher began working with community stakeholders to understand the epidemic of type 2 diabetes in the community [16]. In 2014, the lead researcher worked with CBPR stakeholders to adapt and implement culturally appropriate diabetes self-management education [14,17,18]. While the self-management study continues, preliminary research revealed numerous and significant environmental barriers that constrain self-management behaviors. CBPR stakeholders asked that ecological barriers be documented and discussed. At the request of CBPR stakeholders, this work highlights the environmental barriers faced by Marshallese living in Arkansas.

Diabetes self-management is an evidenced-based method for addressing type 2 diabetes that focuses on patient health behaviors [19]. While diabetes self-management has shown improvement in health outcomes, there is evidence that it is not accessible and effective for all populations [20,21]. Furthermore, diabetes self-management may over-emphasize personal responsibility, giving limited consideration to the community, organizational, and policy environments that also affect health outcomes [22]. Critics of the self-management and personal responsibility paradigm argue, “an overriding emphasis on personal responsibility blames the victim, by ignoring the social context in which the individual decision making and health-related action takes place [23].”

## Theoretical Framework

Diabetes self-management requires access to a variety of resources beyond individual agency, and the Social Ecological Model considers the environmental factors that constrain or facilitate access to organizational, community, and policy resources [23]. The Social Ecological Model brings together multiple fields of research and provides a framework for examining participants’ health in relationship with their natural and social environments. This multi-dimensional focus allows researchers to examine individual-environment interactions that affect access and use of resources and services [24]. Since effective self-management is contingent on both individual and environmental resources, systematic patterns of inequality may emerge at environmental levels.

**Figure 1:**
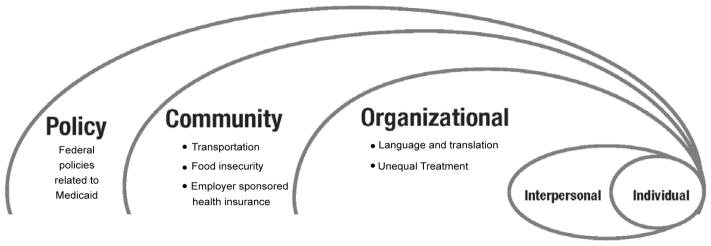
Social Ecology of Marshallese Health

While much research has focused on individual-level barriers to diabetes self-management, an ecological perspective integrates both individual and environmental factors to examine health outcomes of individuals. Ecological models delineate the reciprocal relationships between individual/interpersonal factors and broader organizational, community, and policy determinants of health behaviors [22]. In contrast to perspectives that emphasize personal responsibility for disease self-management, this body of literature highlights the confluence of multiple environmental dimensions of health across the lifespan [24–26]. Examining individual and environmental determinants of health behaviors and outcomes allows for complex comprehension of the social and cultural contexts and situational factors that can affect efforts to manage one’s health [24] (Figure 1).

## Methods

Over a period of two and a half years, the CBPR team conducted several qualitative pilot projects to address diabetes disparities with research questions focused on: general beliefs about health and use of health care services, health beliefs regarding diabetes self-management behavior, and perceptions of family-model diabetes self-management (Table 1).

Twelve focus groups were conducted with Marshallese participants (n = 69). All focus group participants were recruited through CBPR partners: Arkansas Coalition of Marshallese, Gaps in Services to Marshallese Task Force, Marshallese pastors, and the local Republic of the Marshall Islands Consulate in Springdale, Arkansas. The research team worked with CBPR stakeholders to create culturally appropriate study announcements, and community partners provided potential participants with information about the research. All participants provided consent prior to data collection, were age 18 or older, and self-reported as Marshallese. A Marshallese community co-investigator was available at each focus group to provide translation if needed. Focus groups ranged from four to twelve participants. A semi-structured interview guide created by the CBPR team included open-ended questions that allowed participants to speak in-depth about their health beliefs and experiences. All focus groups were conducted in private community rooms to ensure participant confidentiality. The duration of each focus group was approximately one hour. Focus groups were recorded and transcribed verbatim. Participants received a $25 gift card as compensation for their time. Each of the study’s procedures were reviewed and approved by the University of Arkansas for Medical Sciences Institutional Review Board (Table 1).

Each of these projects focused on gathering data to answer study-specific research questions and concentrated on personal/interpersonal beliefs, experiences, and behavior. While research questions centered on describing general beliefs about health, with a focus on individual and interpersonal elements, there were a number of emergent themes that participants discussed during the course of the research projects described above that pointed to larger organizational, community, and policy barriers to health care. As a part of our commitment to a CBPR paradigm, a collective decision was made by the CBPR researchers and stakeholders to ensure participants’ voices were heard regarding these environmental barriers.

Emergent themes were coded based upon the environmental barriers identified by participants. These emergent themes were organized into priori groups according to the Social Ecological Model’s levels: organizational, community, and policy. A codebook was developed with the priori codes and emergent codes (Table 2).

Coding was conducted by four qualitative researchers with two primary coders and two confirmation coders. Researchers collaborated through the course of data analysis to ensure inter-coder reliability. Marshallese community co-investigators provided feedback throughout the research and writing process. The Marshallese community co-investigators’ involvement helps reduce the risk of misinterpretation because of language barriers or cultural nuances in meaning, and helps ensure findings and discussion accurately reflected what Marshallese participants stated.

## Results

The results are presented using the three environmental levels of the Social Ecological Model as an organizing template. The environmental levels are *organizational, community,* and *policy*. First, Marshallese informants report barriers to diabetes self-management at local health care organizations including issues with: (a) language and translation, and (b) unequal treatment. Second, participants report three barriers to diabetes self-management at the community level including: (a) transportation, (b) food insecurity, and (c) employment and employer sponsored health insurance. Finally, participants discuss barriers at the policy level, which is the macro-level of the social ecology of health. Participants identify federal policies related to Medicaid as a barrier to self-management of diabetes.

### Organizational

#### Language and translation

Participants highlight a lack of translated materials (e.g. care instructions, patient forms, and information on medicines) as well as a lack of organizations that hire Marshallese translators. Marshallese participants discuss that “the language barrier is also a barrier to better health.” Participants state that successful diabetes education for the community would include educational materials “translated into (the) Marshallese language.”

Language barriers and the lack of translators affect Marshallese decisions about whether or not to seek care. Participants describe that when Marshallese patients have a doctor’s appointment “they won’t show up because of (the) language barrier.” For the Marshallese, knowledge that they will likely not be provided care with a translator greatly reduces the choices that they have when seeking care, even in instances where they may be adequately covered by insurance.

Another participant notes that these issues go beyond merely providing translation of language, since many Marshallese often need additional translation of medical concepts. One participant states that “every time the receptionist would do the intake for the new patients, she would ask them, ‘Do you have PCP (Primary Care Physician)?’ They’re like—they don’t know what PCP is, cause we don’t have PCPs in our island…they wouldn’t answer, or they just look at her.”

#### Unequal treatment

Participants share concerns about the way that uninsured and underinsured Marshallese patients are treated by area doctors, clinics, and hospitals. Participants speak about the difficulties of receiving care with inadequate levels of insurance coverage. To be clear, Marshallese patients state that they are not waiting to seek medical care until they are at a crisis point, but that organizational policies limit their options. As one of the participants notes, “[A recent study] conclusion is that the Marshallese wait until they are very sick and seek medical attention…that is not true…we go and seek medical attention, (but the doctors and hospitals) don’t give us attention.”

Marshallese participants also discuss their experiences with unequal treatment at doctor’s offices, clinics, and hospitals, which participants believe is based on their insurance status or Marshallese ethnicity. As one participant states:

If they don’t have insurance, they go and see the doctor, [the doctor] always keep them until the last, because they don’t have the insurance. They wait there. They are very sick, and they’ve been waiting for them (the doctor/doctor office staff) to call their names. Finally, they have to go to another place, because they are really sick but they (the doctors) don’t really take care of them.

For some patients, this may result in a last resort visit to the emergency room at a local hospital, where they have access to some form of care. However, participants report that even hospital visits can be fraught with obstructions stemming from insurance status. “It seems like some of the hospitals, they only take those that have insurance, not the ones that didn’t have,” notes one participant.

It is not just uninsured Marshallese that experience barriers to accessing health care. For a number of participants, even when they qualify for Medicaid, because they are US citizens, participants note that there are still issues with their care. One participant explains that after changing from private insurance to Medicaid their “(previous doctor) doesn’t take anyone with Medicaid. A lot of doctors here in the United States are not taking Medicaid patients. They will take the people with good insurance. I mean, you go in there and present your Medicaid card, well, find someone else.”

### Community

#### Transportation

Public transportation is limited in northwest Arkansas, and participants report that transportation issues can make every aspect of diabetes self-management more difficult for Marshallese households. Many participants explain that it is necessary to have a car in order to meet basic needs, including access to employment, health care services, diabetes education, and healthy food. Importantly, access to a car is not the only requirement. Currently, the drivers’ license test and testing materials are not available in Marshallese, and because many Marshallese do not read English well, they cannot pass the drivers’ licensing exam. Without a driver’s license, they have limited access to the doctor, work, pharmacy, and grocery store, and face deportation if they drive without a license. Participants note that:

As you… know that most of the Marshallese are being deported because they keep driving without license because: one, they really want to bring their family needs (food, doctor, employment) and two, pick their children from school. There may be one person in the household who knows how to drive but he or she does not have a license but it is hard to ask somebody to drive for you to pick your child from school and also to get your needs.

#### Food insecurity

A lack of access to adequate and healthy food is another important theme that participants identify as creating barriers to health and to diabetes self-management. Participants frequently say that “when (we) are educated… some of (us) will try to change what (we) eat, but the problem too is the cost of vegetables.” Participants state that the affordability and ability to store and prepare healthy foods is an issue that cannot be addressed merely through diabetes education and self-management practices, which often assume that dietary choices are unfettered by cost and other constraints. “At the beginning of the week, I have healthy food, but after a few days, I don’t have money to buy vegetables and meat and fruit. Then it is just Ramen.” While traditional self-management programs recommend switching from sugar to the use of products, such as Splenda, there are issues where “most Marshallese … make little more than minimum wage and if you are going to buy Splenda compared to like regular sugar, the regular sugar is cheaper than (other options),” according to participants.

#### Employment and employer sponsored health insurance

Participants explain that their access to health care is constrained by the kinds of employment that the community can access. Participants state that most of the Marshallese are in jobs that pay low wages and are part-time, or have varying weekly hours, and this means they have to make the choice between everyday necessities, like food, shelter, and insurance premiums.

Even when employers offer health insurance plans, participants find these plans to be too costly for many families, even when these plans were partially subsidized by an employer. “The reason why most Marshallese don’t buy in (to employer insurance plans) at work is because they can’t afford it.”

The prevalence of chronic illnesses in the community is confounded by issues with employer sponsored insurance. Marshallese focus group participants state that

(Marshallese people) don’t have any insurance because they don’t work for some reason… They disqualified to be employed (due to health). That’s not when you go to the employer, they will ask—easily check. Then (employers) will say, “Oh, you are sick. You are sick, you cannot do the job.” That’s why most of the Marshallese been laid off from work, because of their ill condition.

Participants describe the lack of ability (or the employer’s perception of employee’s abilities) to perform job duties means that they cannot work. Participants also state that this renders insurance coverage beyond the financial capabilities of Marshallese households because employer sponsored health insurance is not an option if you cannot get hired. Lack of insurance limits a person’s ability to manage chronic illnesses, therefore limiting their ability to become healthy enough for employment.

One Marshallese elder states that due to the health issues they developed as a result of nuclear radiation exposure they had to quit their job, and then could not afford their insurance premiums.

I was afraid that I’m gonna bleed while I’m on the line… [so], I would go into the bathroom every 20 minutes to take care off that. I quit working. They told me that I was eligible for social security or disability or whatever, and I’ve been trying to apply…I turned 60 yesterday. I don’t have nothing. I lost my insurance. Whenever I quit working, my insurance went up to $400.00 a month, which I couldn’t afford. I dropped all that.

The precariousness of a full-time designation for some employees can make access to insurance programs fleeting. One participant describes that “now that they already cut (hours of employment) and I don’t have (work for) 8 hours. But work 4 hours. I lost my insurance.” Participants state that the difficulty of maintaining health coverage constrains their ability to manage their disease.

### Policy

Current federal health policy limits access to insurance for the Marshallese COFA migrants residing in the United States. The passage of the Personal Responsibility and Work Opportunity Reconciliation Act of 1996 (PRWORA) made COFA migrants ineligible for federally-funded benefits programs, including Medicaid. PRWORA excluded COFA migrants from the category of “qualified immigrants [27].” Despite this, COFA migrants are still required to carry insurance through the Affordable Care Act (ACA). COFA migrants do qualify for income-dependent subsidies. However, these subsidies are based upon a higher income level than most Marshallese have and may not be enough to place insurance premiums within the financial means of most Marshallese households.

Throughout each focus group, participants discuss the lack of access to Medicaid and Medicaid Expansion. They stated that because of the lack of access to Medicaid and Medicaid Expansion under the ACA, many Marshallese residents of the United States do not have access to adequate insurance coverage or health care.

Focus group participants note that there are very few Marshallese who have consistent access to insurance, with some Marshallese estimating that only 20% to 30% of the community are covered by some form of insurance. This is discussed as a life-or-death issue for members of the community. One participant identifies that “we Marshallese have to figure out a way to better our lives because most of us have no medical insurance to seek medical care and I am one that have real high diabetes, I think I would have died (without some access to care).”

## Discussion

The Marshallese community suffers from diabetes at rates that are more than 400% higher than the general US population [4–6], and the disease is poorly managed within the population. Most intervention efforts to improve diabetes self-management behaviors are focused on individual behaviors (individual level) [28]. Much research examines the efficacy of self-management interventions, and diabetes self-management education results in improved health outcomes and reduced costs [29], but improvements are not achieved equally in all populations [30]. The Social Ecological Model provides a framework for understanding environmental elements that affect a person’s health at the organizational, community, and policy level. There is emerging evidence that ecological factors at the organizational, community, and policy levels can create systems of structural inequality for minority and low socioeconomic communities [31].

Through this research, participants identify barriers at organizational, community, and policy levels that constrain efforts to achieve diabetes self-management. For example, patients are instructed to eat nutritionally dense food and avoid processed carbohydrates and fatty meats. However, because the Marshallese are often employed at low-wage jobs, and one worker often supports many in the household, they cannot afford healthy foods. The foods they can afford and the ones provided at local food pantries are primarily processed carbohydrates and fatty, canned meats. To self-manage diabetes, patients are instructed to see their doctor regularly, take their medications as prescribed, and monitor their glucose twice per day. However, exclusion from Medicaid means many Marshallese are left without insurance. The lack of insurance means they cannot afford to see the doctor, purchase their medication, or obtain glucometer strips. Because of these barriers, many Marshallese patients report that they receive sporadic care. This lack of care may result in emergency room visits when chronic conditions reach a severe state.

Transportation issues, such as the lack of public transportation, the necessity to purchase a car, and the need to obtain a driver’s license, affect all areas of self-management. Even for households that have access to a car, the driver’s license test is not offered in Marshallese, and many Marshallese drivers are unable to pass the test. This leaves entire households without transportation to go to the store for healthy food or go to the doctor and pharmacy.

Language barriers remain for those who overcome transportation and insurance barriers. Marshallese speakers are often unable to understand treatment instructions because there are no staff members who speak Marshallese to translate the doctor’s orders, and patient materials are also not provided in Marshallese. The participants also perceive their treatment by health care providers as being discriminatory and unequitable.

Employment issues for the Marshallese include limited access to both full-time employment and access to employer-subsidized insurance programs. Marshallese households experience barriers to health care due to part-time employment and variable weekly hours. This precludes workers, and their extended families, from accessing employer subsidized insurance, and from having funds necessary to pay for healthy food, doctors’ visits, or medication.

These ecological factors create a web of barriers that prevent Marshallese diabetes self-management behavior. It is evident that to achieve improvement in diabetes self-management, we must move beyond individual-level interventions and address barriers at the community, organizational, and policy level.

### Recommendations for practice and policy

At the organizational level, health care providers can offer translated patient materials in the Marshallese language and hire Marshallese health workers to provide oral translation. At the same time, it is important for health professionals to ensure culturally appropriate translations of medical concepts. Additionally, local health care providers should conduct internal reviews of policies regarding Marshallese patient experiences to find ways to improve patient care and experiences. Health care providers should also provide all employees with Marshallese cultural awareness training.

To address community level barriers, the inadequacy of transportation and healthy food options in the area must be addressed. It is necessary that local communities invest in public transit. In addition, agencies responsible for drivers’ licensing and vehicle registration should translate policies and materials into Marshallese. Food pantries should implement healthy food donation polices, which would allow them to distribute healthier food choices. Employer-subsidized insurance plans should be made more accessible to Marshallese employees through increased options for consistent, full-time status. In addition, employers should also consider increasing insurance subsidies to ensure that Marshallese employees can afford insurance plans. Full-time employment would also provide other benefits to the community, such as income to purchase healthy foods and necessary medications for the household. Additionally, providing more education to employees concerning their health benefits, particularly translated into Marshallese, is also essential to facilitate Marshallese access to these programs.

At a policy level, Medicaid should be restored for COFA migrants through amendment of PRWORA to include them within the category of qualified immigrants. Marshallese were eligible for Medicaid when they agreed to the COFA; however, they were left out when PRWORA’s definition of qualified immigrants did not include COFA migrants [32]. Bills have been introduced in the US House of Representatives and the US Senate to restore Medicaid coverage for COFA migrants lawfully residing in the United States by amending Title IV of the PRWORA. If approved, this would restore Medicaid for the Marshallese and provide other eligible COFA migrants with Medicaid (Table 3).

### Limitations and Strengths

The data for this study was derived from numerous focus groups where the primary purpose was the elicitation of information related to personal and interpersonal level beliefs and barriers to health care and diabetes self-management. The fact that these environmental level concerns emerged is both a strength and a limitation to the design. While the studies were not constructed to elicit information about the environmental context of health and diabetes self-management, the CBPR approach requires that we disseminate the full results of the focus groups. This study allows the voices of participants and their lived experiences to be documented and published. An additional limitation is the convenience sample, which reduces the generalizability of the results. Despite these limitations, this study provides insight into the environmental barriers to diabetes self-management for the Marshallese and provides recommendations for addressing those barriers.

## Conclusion

Using the Social Ecological Model as a framework, this research provides an analysis of Marshallese health that expands the traditional diabetes self-management perspective. Understanding of environmental barriers is necessary to support patients in their self-management efforts. Researchers, health care providers, and policy makers have the challenge of addressing these environmental barriers, along with providing culturally appropriate self-management education in their efforts to reduce diabetes disparities within the Marshallese population.

## Figures and Tables

**Table 1:** Studies from which the data was drawn

Focus Group Topic	Number of focus groups	Total Number of Participants	Dates
General beliefs about health and use of health care services	2	Total of 15 participants	November 2013
Health beliefs regarding diabetes self-management behavior	5	Total of 26 participants	March 2014–April 2015
Perceptions of family-model diabetes self-management	5	Total of 28 participants	June 2014–September 2014

**Table 2:** Themes and sub-themes

Priori Groups	Emergent
Organizational	Language and translation Unequal treatment
Community	Transportation Food insecurity Employment and employer sponsored health insurance
Policy	Federal policies related to Medicaid

**Table 3:** Recommendation for Policy and Practice

Social Ecological Model Levels	Barriers	Policy/Practice Recommendations
Organizational	Language and translation Unequal treatment	Translate patient materials and utilize translators Hiring Marshallese staff as community health workers Ensure culturally appropriate translations of medical concepts Conduct internal reviews of policies Provide cultural awareness training to employees
Community	Transportation Food insecurity Employment and employer sponsored health insurance	Invest in public transit Translate driver’s license testing material Implement health food policies at food pantries Provide more full-time employment Ensure employees understand their insurance benefits
Policy	Federal policies related to Medicaid	Amend PRWORA to include COFA migrants within the category of qualified Immigrants
